# Differential miRNA Profiling Reveals miR-4433a-5p as a Key Regulator of Chronic Obstructive Pulmonary Disease Progression *via* PIK3R2-mediated Phenotypic Modulation

**DOI:** 10.2174/0113862073243966231030093213

**Published:** 2024-01-04

**Authors:** Siming Tao, Chunyan Liao, Yide Wang, Dan Xu, Zheng Li, Fengsen Li

**Affiliations:** 1 Department of Respiratory and Critical Care Medicine, Fourth Affiliated Hospital of Xinjiang Medical University, Urumqi, China;; 2 Xinjiang Laboratory of Respiratory Disease Research, Traditional Chinese Medicine Hospital Affiliated to Xinjiang Medical University, Urumqi, China

**Keywords:** COPD, progressive development, miRNA, dual-luciferase reporter assay, cell transfection, biological phenotypes

## Abstract

**Objective:**

In this study, a high-throughput sequencing technology was used to screen the differentially expressed miRNA in the patients with “fast” and “slow” progression of chronic obstructive pulmonary disease (COPD). Moreover, the possible mechanism affecting the progression of COPD was preliminarily analyzed based on the target genes of candidate miRNAs.

**Methods:**

The “fast” progressive COPD group included 6 cases, “slow” and Normal progressive COPD groups included 5 cases each, and COPD group included 3 cases. The peripheral blood samples were taken from the participants, followed by total RNA extraction and high throughput miRNA sequencing. The differentially expressed miRNAs among the progressive COPD groups were identified using bioinformatics analysis. Then, the candidate miRNAs were externally verified. In addition, the target gene of this miRNA was identified, and its effects on cell activity, cell cycle, apoptosis, and other biological phenotypes of COPD were analyzed.

**Results:**

Compared to the Normal group, a total of 35, 16, and 7 differentially expressed miRNAs were identified in the “fast” progressive COPD, “slow” progressive COPD group, and COPD group, respectively. The results were further confirmed using dual-luciferase reporter assay and transfection tests with phosphoinositide- 3-kinase, regulatory subunit 2 (*PIK3R2*) as a target gene of miR-4433a-5p; the result showed a negative regulatory correlation between the miRNA and its target gene. The phenotype detection showed that the activation of the phosphatidylinositol 3 kinase (PI3K)/protein kinase B (AKT) signaling pathway might participate in the progression of COPD by promoting the proliferation of inflammatory A549 cells and inhibiting cellular apoptosis.

**Conclusions:**

MiR-4433a-5p can be used as a marker and potential therapeutic target for the progression of COPD. As a target gene of miR-4433a-5p, PIK3R2 can affect the progression of COPD by regulating phenotypes, such as cellular proliferation and apoptosis.

## 1 INTRODUCTION

Chronic obstructive pulmonary disease (COPD) is a smoking- and age-related disease**-**mainly characterized by persistent respiratory symptoms and restricted airflow. When COPD occurs, the patient's lung function cannot be completely reversed and progressed. However, the progressive development of lung function in the different types of patients is significantly different. The specific clinical diagnosis and treatment showed two types of phenotypes with significant differences [1, 2], including “fast” disease progression, having a short course of the disease, but rapid deterioration of pulmonary function level and systemic respiratory symptoms, and “slow” disease progression, having a prolonged disease course, but relatively stable pulmonary function level and systemic respiratory symptoms. Based on large early-stage clinical data, our team developed criteria for detecting the “fast” and “slow” progression of COPD and improved their diagnostic criteria using the “Delphi method” simultaneously [1, 2].

Currently, the markers for monitoring COPD essentially include blood-related indicators, such as inflammation, enzymes, anti-protease, and oxidative stress-related biomarkers. Due to the difficulty in obtaining lung tissues, clinical studies based on lung tissues are not convenient. Over the past years, numerous studies have been performed on COPD-related biomarkers, among which studies on miRNA are important [3]. miRNA can be obtained from blood and detected rapidly, favoring the developmental direction of clinical studies; moreover, miRNA is not only used for COPD but also for oncogenic virus-driven malignancies, such as lymphoma. Therefore, it might also become a new focus of prognosis prediction and individualized treatment of the disease [4-7].

This study aimed to explore the specific differential markers between the two extreme phenotypes of “fast” and “slow” progression of COPD and provide a theoretical basis for the treatment and prognosis evaluation of COPD from the perspective of its progression speed. Additionally, an inflammatory cell model of COPD was established *in vitro* to verify the effects of overexpression or inhibition of candidate miRNA and target gene on cellular activities, cell cycle, and cellular apoptosis. Moreover, complete dual-luciferase reporter assay and cell transfection experiments were performed to analyze and observe the effects of candidate miRNA on disease phenotype.

## 2 MATERIALS AND METHODS

### 2.1 Screening and Verification of miRNA Molecular Markers

#### 2.1.1 Subjects

A total of COPD patients and healthy participants, who were admitted to the Fourth Affiliated Hospital of Xinjiang Medical University from January 2017 to May 2021, were recruited for this study.

#### 2.1.2 Diagnostic Criteria

In this study, COPD was diagnosed using Global Initiative for Chronic Obstructive Lung Disease (GOLD) guidelines (2017 revised). The diagnostic criteria for the “fast” progression of COPD were as follows: necessary conditions included duration of disease ≤5 years and pulmonary function grade ≥III; secondary conditions included the number of acute exacerbations ≥3 times per year, number of hospitalizations due to disease exacerbations ≥2 times per year, modified British medical research council (mMRC) ≥grade 3, COPD Assessment Test (CAT) score >20 points, and body mass index (BMI) <21. The presence of two of the secondary conditions was sufficient for diagnosing COPD's “fast” progression. The diagnostic criteria for the “slow” progression of COPD were as follows: necessary conditions included duration of disease ≥15 years and pulmonary function ≤II; secondary conditions included the number of acute exacerbations ≤2, number of hospitalizations due to disease exacerbations ≤1, mMRC ≤grade 2, CAT score ≤20 points, BMI ≥21. The presence of two of the secondary conditions was sufficient for diagnosing the “slow” progression of COPD.

#### 2.1.3 Inclusion and Exclusion Criteria

Inclusion criteria: The criteria for the inclusion of the participants in this study were as follows:

1. The patients with ages ranging from 35 to 80 years met the diagnostic criteria of COPD and had no acute exacerbation of COPD within at least 4 weeks.
2. The patients met the diagnostic criteria of “fast” and “slow” development of COPD.
3. The outpatient or community patients and normal healthy persons who signed informed consent.

#### 2.1.4 Exclusion Criteria

The criteria for the exclusion of the participants from this study were as follows:

1. The patients with severe respiratory failure (PaO_2_ <40 mmHg, PaCO_2_ >90 mmHg), pulmonary heart diseases, and pulmonary encephalopathies.
2. The patients who had other respiratory-related diseases, such as bronchiectasis, diffuse panbronchiolitis, lung cancer, pulmonary tuberculosis, and interstitial lung disease.
3. The patients with active tumors, long-term use of the hormone, or immune inhibitor.
4. The patients who could not accurately answer the questionnaire or describe the degree of disease.

#### 2.1.5 Sample Collection

A 2-3-mL peripheral blood sample was collected from each participant into the PAX-gene Blood RNA Tube. After gradient cooling, the samples were stored at –80°C.

#### 2.1.6 miRNA Extraction and Quality Assessment

Total RNA was extracted from the blood samples using a TRIzol reagent. The purity and concentration of extracted RNA were detected using Nanodrop (Optical Density (OD)_260/280_ ≥1.8; OD_260/230_ ≥ 1.0) and Qubit 2.0 (total RNA concentration ≥250 ng/µL), respectively. The integrity of the RNA sample was detected using Agilent 2100 bioanalyzer to ensure that samples qualified for sequencing (RNA integrity number of total RNA ≥8.0, 28S/18S ≥1.5; the baseline of the map was not raised; the 5S peak was normal). Beijing Baimaike Biotechnology Co., Ltd. performed the high-throughput sequencing of miRNA through the technical service contract, which mainly included several essential procedures of miRNA isolation and purification, library establishment, and sequencing.

#### 2.1.7 Analysis of Differential Expression of miRNAs

The low-quality adapter reads, including primer and vector sequences, were filtered out from the data in the original Fastq file to obtain the average value of clean reads. Meanwhile, the Trusted Platform Module algorithm was used to normalize the expression levels of miRNA. The inter-group differential expression analysis was performed using the DESeq method to obtain the miRNA set having differential expression levels between the two biological conditions. In detecting differential expression levels, |log2(FC)| ≥1 and *P* <0.05 were used as the standard criteria for screening differentially expressed miRNAs.

#### 2.1.8 Prediction of Target Genes

miRNAs and their target genes are usually not completely complementary. miRNA mainly interacts with its target gene 3’ untranslated region (3’UTR) and inhibits its transcription and translation. The genes targeted by the differentially expressed miRNAs were predicted using miRanda and RNA hybrid.

#### 2.1.9 Gene Ontology (GO) Enrichment Analysis

GO database is a standard biological annotation system with a clear structure. The GO enrichment of differentially expressed miRNA target genes can describe the affected secondary functions. This enrichment analysis was used for each differentially expressed miRNA with a corresponding *P*-value. If the *P*-value was < 0.05, the differentially expressed miRNA target genes had significant enrichment in the GO.

#### 2.1.10 Analysis of KEGG (Kyoto Encyclopedia of Genes and Genomes) metabolic pathways

KEGG is a functional database about signaling pathways and has advanced biological functions. The database contains the pathway maps of cellular and organism functions, which help in screening the diversity of functional long non-coding RNA (lncRNA) in COPD. The KEGG database was used to identify and confirm the functional pathways of COPD and its respective regulated lncRNAs. Moreover, the signaling pathways significantly enriched by the candidate target genes were summarized, and the metabolic map of each enriched pathway was presented.

#### 2.1.11 Verification using qRT-PCR

Based on the differentially expressed miRNAs identified in the miRNA sequencing results and their target gene functions, five miRNAs related to the two progression phenotypes of COPD were selected for the subsequent validation. TaqMan Advanced miRNA assays were used for the qRT-PCR analysis. The analysis was performed in five steps, including tailing, ligation, reverse transcription, pre-amplification, and PCR. The system and procedures were performed following the manufacturer’s instructions for the reagent kit. The PCR reaction condition included activation at 50°C for 2 min and 40 cycles of denaturation at 95°C for 3 s and annealing/extension at 60°C for 30 s. Each sample was run in duplicates, and the relative differential expression levels of miRNAs in the COPD group were identified in comparison to those of the control group using the 2^-△△CT^ method.

### 2.2 Functional Analysis of Candidate miRNAs and Clinical Re-verification of Target Genes

#### 2.2.1 Subjects

In this experimental section, the subjects included candidate genes targeted by miR-4433a-5p and A549 inflammatory cell model. A549 cells were purchased from Procell Life Science & Technology Co., Ltd. under item number XF9701-A.

#### 2.2.2 Research Scheme

(1) Experimental procedure: The A549 cells were divided into six groups: Group A included A549 cells without any treatment; Group B included A549 cells treated with 1.25% cigarette smoke extract (CSE); Group C included A549 cells treated with 2.5% CSE; Group D included A549 cells treated with 5% CSE; Group E included A549 cells treated with 10% CSE; and Group F included A549 cells treated with 20% CSE. (2) Preparation of smoke reagent. (3) Experimental method: The 293T cells were divided into four groups: Group A included 293T cells transfected with PmirGLO expression vector; Group B included 293T cells transfected with pmirGLO-PIK3R2-WT + hsa-miR-4433a -5p expression vector; Group C included 293T cells transfected with pmirGLO-PIK3R2 (MUT) + hsa-miR-4433a-5p expression vector; and Group D included 293T cells transfected with pmirGLO-PIK3R2 (MUT) + mimics NC expression vector. (2) Preparation of transfection complex. (3) Detection of dual-luciferase.

### 2.3 Statistical Analyses

All the collected data were statistically analyzed using GraphPad Prism 8.0. Each experiment was performed in triplicates. The measured data were expressed as mean ± standard deviation. The independent sample *t*-test was used for the comparison of the two groups.

### 2.4 Patients and Public Involvement

The patients or the public were not involved in the designing, conducting, reporting, or dissemination plans of this study. The specific research process of this study is shown in Fig. (**1**).

## 3 RESULTS

### 3.1 Clinical Results

A total of 263 participants (188 males and 7 females), including 64 patients with “fast” progression of COPD, 155 patients with “slow” progression of COPD, 21 COPD patients, and 23 healthy individuals, were recruited for this study. To exclude the effects of smoking factors, five men with smoking habits were selected from each of the “slow” progression of COPD and Normal groups, three men with smoking habits were selected from the COPD, and six men with smoking habits were selected from the “fast” progression of COPD. The DNA sample extracted from one sample in the Normal group was unqualified; therefore, the COPD group contained four samples. There were negligible differences in the ages and genders among the four groups (*P* >0.05, Table **1**).

### 3.2 Differential Expressions of miRNAs

**Fig. (2):**
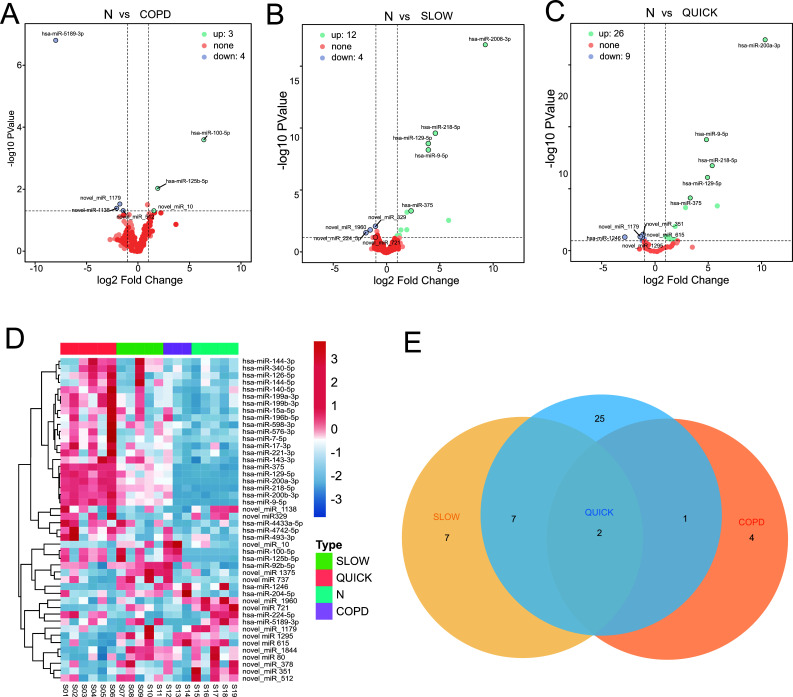
Differential analysis between the Normal group and “fast” and “slow” progression of COPD groups. **Notes: (A)** Volcanic maps of miRNA differences between the COPD group and control group; **(B)** Volcanic maps of miRNA between the slow COPD group and the control group; **(C)** Volcanic maps of miRNA between the fast COPD group and the control group; **(D)** Heat maps of different miRNAs between the three groups; **(E)** Wayne diagram of differential miRNAs between the three groups.

DESeq method was used to detect the differentially expressed miRNAs in the progressive development of COPD according to the standard of log2 ratio ≥1 and *P* ≤0.001. The results showed that, as compared to the Normal group, a total of 47 differentially expressed miRNAs were identified in the COPD patients, indicating that these miRNAs might have significant regulatory effects on the occurrence of COPD. Sixty differentially expressed miRNAs were identified among the two progressive groups, suggesting that these miRNAs might have important regulatory effects on the progressive advancement of COPD. A thorough screening revealed that 47 differentially expressed miRNAs, which could regulate the occurrence of COPD, could also regulate the progressive development of COPD (Fig. **2**).

As shown in Fig. (**2A****-****2C**), drawing the volcano plots of differentially expressed miRNAs in the three groups identified three upregulated miRNAs and four downregulated miRNAs in the COPD group; 12 upregulated miRNAs and four downregulated miRNAs in the “slow” progression of COPD group; and 26 upregulated miRNAs and nine downregulated miRNAs in the “fast” progression of COPD group. Figs. (**2D** and **2E**) show the heatmap and Venn diagram of the differentially expressed miRNAs in the three groups.

### 3.3 Network Prediction of the Genes Targeted by Differentially Expressed miRNAs

The original RNA-seq data of 32 genes (GSE38974) was downloaded from the GEO database for the identification of differentially expressed genes (DEGs), which were combined with the differentially expressed miRNAs obtained in the three groups for the prediction of the regulatory network.

Fig. (**3A**) shows the heatmap of DEGs, including 240 and 232 downregulated and upregulated genes, respectively, in the GSE38974 dataset, which were identified using the R package “DESeq2” with threshold |FC| >2 and *P* <0.05. Fig. (**3B**) shows the sequence binding prediction of DEGs and differentially expressed miRNAs performed using miRanda. The genes were identified by predicting their 3’-UTR sequence. There were 43 miRNAs, 316 genes, and 1608 relational pairs, as shown in Fig. (**3B**).

### 3.4 Results of GO Enrichment Analysis

**Fig. (4):**
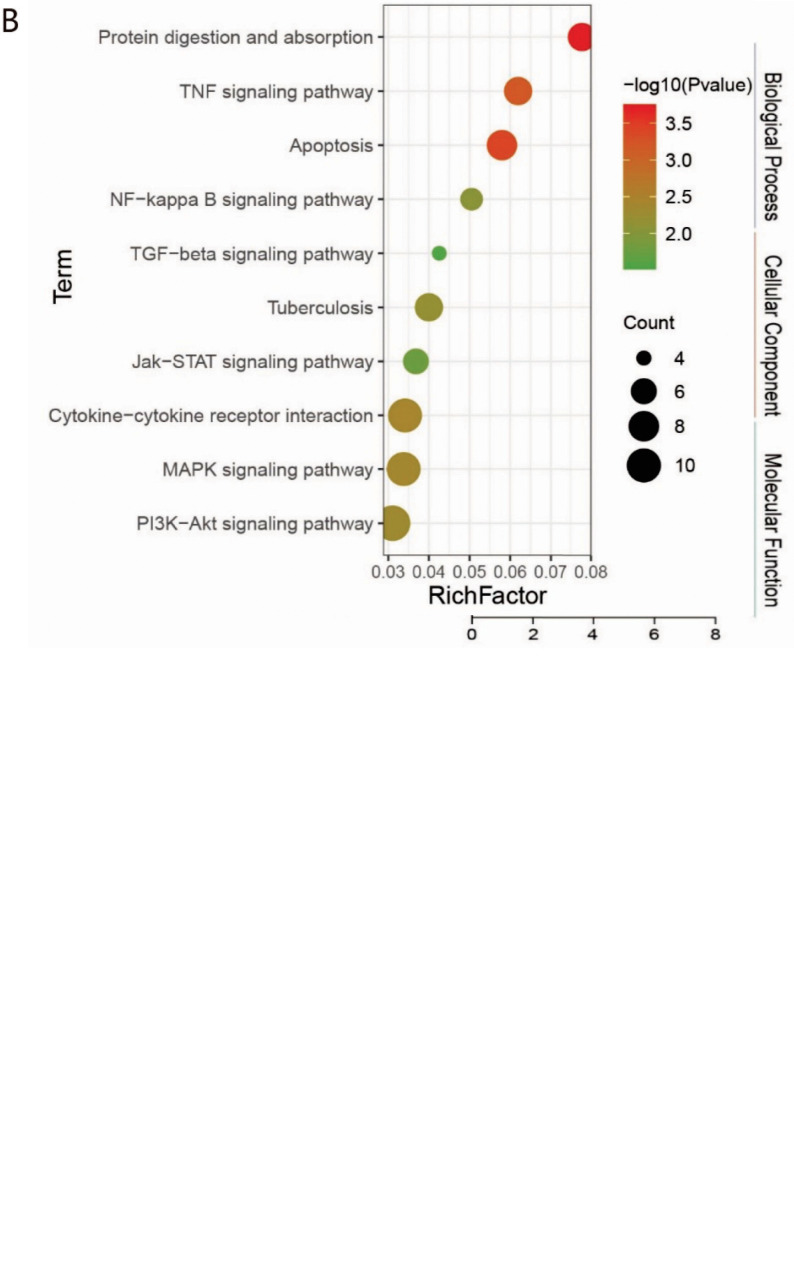
GO and KEGG pathways enrichment analyses. **Notes: (A)** GO enrichment analyses of differential genes. **(B)** KEGG pathways enrichment analyses of differential genes.

The GO enrichment analysis showed that the genes targeted by the differentially expressed miRNA in the COPD patients were mainly enriched in biological processes, such as cellular processes, single species processes, and biological regulation. The differentially expressed miRNA might regulate the differentiation of the muscle system and Treg cells. The cell component enrichment analysis showed that these genes were mainly enriched in extracellular matrix-containing collagen, endoplasmic reticulum, and cationic complex of adenosine-triphosphate transport by affecting the synthesis of collagen, glycosaminoglycan, and heparin (Fig. **4A**).

The KEGG pathway enrichment analysis showed that the top ten enriched signaling pathways included protein digestion and absorption, tumor necrosis factor (TNF), apoptosis, nuclear factor-kappa B (NF-κb), TGF-β (transforming growth factor-β), tuberculosis-related, Janus kinase-signal transducer and activator of transcription (JAK-STAT), cytokine, Mitogen-activated protein kinase (MAPK), and phosphatidylinositole-3-kinases-protein kinase B (PI3K-AKT) signaling pathways. Most of the signaling pathways were related to immune inflammation, while many were closely related to COPD studies (Fig. **4B**).

### 3.5 Verification Using qRT-PCR

**Fig. (5):**
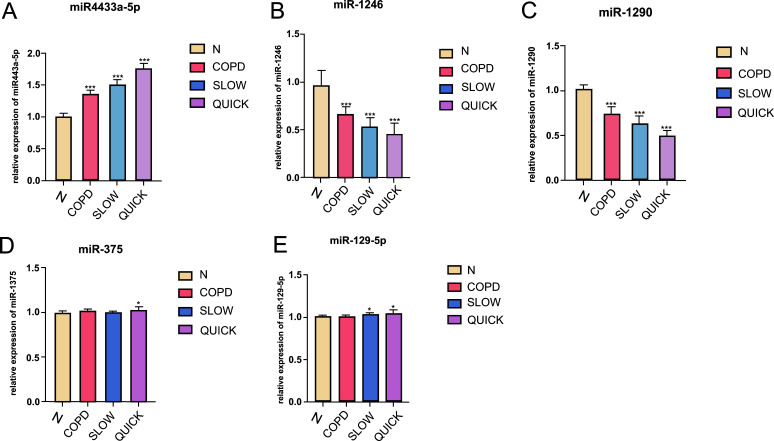
qRT-PCR results of the selected miRNAs. **Notes: (A)** The differential expression of miR4433a-5p between groups; **(B)** The differential expression of miR-1246 between groups; **(C)** The differential expression of miR-1290 between groups; **(D)** The differential expression of miR-375 between groups; **(E)** The differential expression of miR-129-5p between groups.

A total of five miRNAs with relatively small *P-*values, including hsa-miR-1246, hsa-miR-4433a-5p, hsa-miR-1290, hsa-miR-375, and has-miR-129-5p, which might play a role in the pathogenesis of COPD, were selected. A total of 60 individuals, including 15 from each of the “fast” progression, “slow” progression, COPD, and Normal groups were selected to verify the reliability of high-throughput sequencing data. The results demonstrated that miR-4433a-5p had a high expression in the COPD group, which gradually increased with the increase in the progression of COPD, as shown in Fig. (**5A**). Moreover, the miR-1246 and miR-1290 had low expression in the COPD group, which gradually decreased with the decrease in the progression of COPD (Figs. **5B** and **5C**), while the expression levels of hsa-miR-375 and hsa-miR-129-5p did not show significant differences among the groups (Figs. **5D** and **5E**).

### 3.6 MTT Detection Using the Smoke Inflammatory Cell Model

**Fig. (6):**
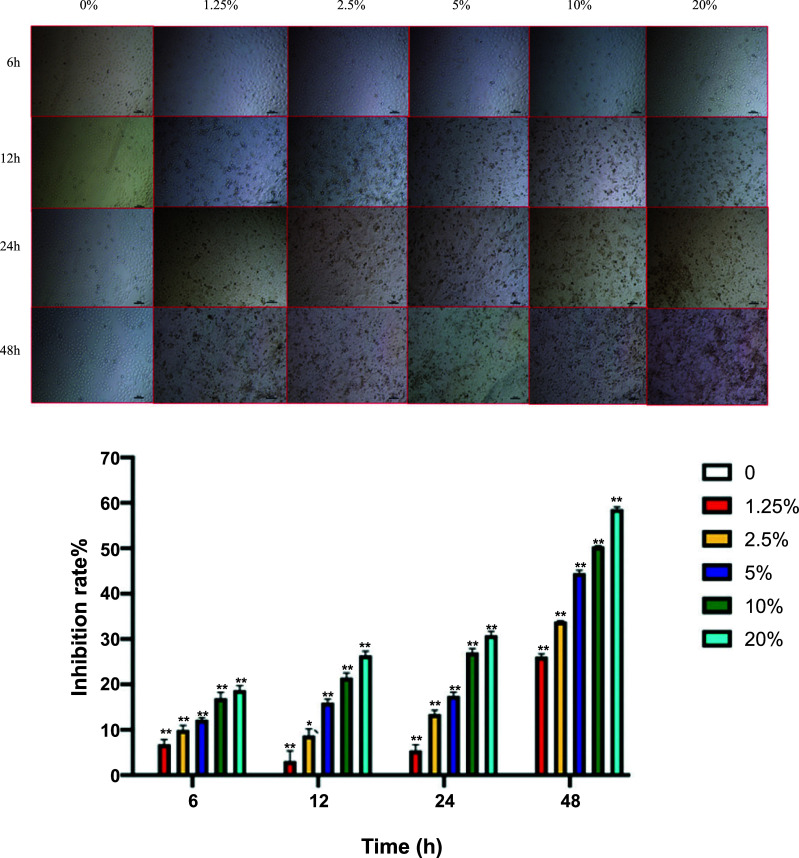
Correlation analysis of CSE concentration and proliferation inhibition of A549 cells over time. **Notes:** This picture includes both a visual display and a quantitative analysis of the bar chart display.

The MTT detection revealed similar changing trends in the groups at each time point. Compared to the control group, the CSE treatment with different concentrations inhibited cellular proliferation to different degrees in a dose-dependent manner. After 6 h, 12 h, 24 h, and 48 h, the treatment of A549 cells with different concentrations of CSE could cause different degrees of inhibition, which increased with the increase in concentration. The general inhibition model referred to IC50, which not only achieved the purpose of cell damage but also caused serious damage to the cells, which mainly depended on the morphology of cells. The cells became dull, shrank, and had no unfolded morphology. Therefore, based on these results, the 24 h treatment duration and 10% CSE treatment concentration were selected (Fig. **6**).

### 3.7 Dual-luciferase Reporter Assay, Target Gene Analysis, and Biological Phenotype Detection of Transfection Test

Using dual-luciferase reporter assay, the genetic testing results showed that the co-transfection of PmirGLO-*PIK3R2* (WT) and hsa-miR-4433a-5p mimics in 293T cells significantly decreased the luciferase activity. In contrast, the transfection of PmirGLO-*PIK3R2* (MUT) showed no difference as compared to the groups. These results suggested that hsa-miR-4433a-5p could negatively target the *PIK3R2*, as shown in Fig. (**7A**). The RT-PCR results showed that, after transfecting mir-4433ap mimics, the expression level of miR-4433a-5p increased and that of its target gene *PIK3R2* decreased significantly as compared to the negative control group. The treatment of cells with a miR-4433a-5p inhibitor decreased the expression of miR-4433a-5p, which led to an increase in the expression level of its target gene *PIK3R2* compared to the control group. The CCK8 detection showed that as compared to the A549 group, the proliferation of the A549 cells treated with CSE and transfected with mimiR4433a mimic significantly increased, while the proliferation rate of those treated with CSE and mimiR4433a inhibitor decreased significantly, as shown in Figs. (**7B****-****7D**). Western blot analysis revealed that, as compared to the control group, the expression level of PIK3R2 significantly downregulated and upregulated in the A549 cells transfected with hsa-mir-4433ap mimics and miR-4433a-5p inhibitors, respectively, as shown in Fig. (**7E**). compared with the control group, the expression of PIK3R2, which is a target gene of the A549+CSE group, was significantly down-regulated. The knockdown of PIK3R2 in the A549 cells treated with CSE significantly downregulated the expression level of the target gene, as shown in Figs. (**7F****-****7H**). Moreover, the streaming apoptosis results revealed that, compared to the control group, the cellular apoptosis rate decreased and increased significantly in the miR4433a-5p mimic and miR4433a-5p inhibitor groups, respectively, as shown in Figs. (**7I** and **7J**).

## 4 DISCUSSION

COPD is one of the three major causes of chronic disease-related deaths worldwide. In China, the prevalence of COPD over 40 years has been as high as 13.7% [8]. Previous studies by our research group analyzed and summarized the clinical characteristics of COPD patients in the early stage using the Delphi method and reported two extreme clinical phenotypes [1, 2], including the “fast” and “slow” progression of COPD. Similarly, the clinical phenotype of rapidly progressive interstitial lung disease (PF-ILD) has recently been proposed [9, 10], providing a better research clinical phenotype for the clinical treatment and follow-up of ILD patients. Using “post gene” omics, Bird *et al.* reported that the prevention and treatment of diseases from the perspective of epigenetics were the main research direction in the future [11-13]. Currently, numerous studies have been conducted on miRNAs in COPD patients, including studies on clinical phenotype [14-17], diagnosis [18], treatment, and smoke exposure [19-30].

In this study, 47 differentially expressed miRNAs were screened out as compared to the healthy population, and 60 differentially expressed miRNAs were screened out among the different progressive COPD diseases. By screening and comparing these miRNA groups, 8 miRNAs related to different progressive COPD diseases were identified. These miRNAs included miR-1246, miR-375, miR-218-5p, miR-4433a-5p, miR-9-5p, miR-129-5p, miR-5189-3p, and miR-200a-3p. These results were verified using qRT-PCR among 60 participants, including 15 from each of the four groups. The results showed that the expression trend of miR-4433a-5p in the different progressive COPD groups gradually increased, while those of miR-1246 and miR-1290 gradually decreased. Therefore, it was speculated that miR-4433a-5p might be a biomarker or potential therapeutic target for COPD's-”fast” progression.

*PIK3R2* was identified as a target gene of miR-4433a-5p using bioinformatics software. Then, the vector of the target gene was constructed and transfected again. The results showed a targeted binding between the miR-4433a-5p and mRNA of *PIK3R2*. PIK3R2 is an important component of the phosphoinositol-3 kinase (PI3K) family. The *PIK3R2* gene is located on human chromosome 19 q13 arm and contains 16 exons with a total gene length of 173,346 kb. The protein, which controls the transcriptional synthesis, is mainly distributed in the subunits in the cellular regulatory processes and produces a special enzyme - 3-phosphoinositidase on its plasma membrane [31]. The transfection of miR-4433a-5p mimic and inhibitor was performed in the COPD inflammatory cell model.

The qRT-PCR analysis confirmed that miR-4433a-5p was highly expressed after the transfection of hsa-mir-4433a-5p mimics, while the expression level of *PIK3R2* decreased significantly. After transfecting the cells with the hsa-mir-4433a-5p inhibitor, the expression level of miR-4433a-5p decreased, while that of *PIK3R2* significantly increased. Meanwhile, Western blot analysis showed that, as compared to the control group, the expression level of PIK3R2 decreased significantly after the transfection of hsa-mir-4433a-5p mimics. In contrast, after the transfection of the hsa-mir-4433a-5p inhibitor, the expression level of PIK3R2 increased significantly. Therefore, miR-4433a-5p and its target gene *PIK3R2* might have a negative regulatory correlation. In the miR4433-5p transfection mimics group, the cellular apoptosis rate in the COPD inflammatory cell model decreased significantly, while that in the miR4433a-5p inhibitor group significantly increased. These results were consistent with those of previous clinical studies. The over-expression of miR4433a-5p might inhibit the PI3K/Akt signaling pathway by targeting *the PIK3R2* gene, promoting apoptosis, and improving the inflammatory level of cells. As the PI3K/Akt signaling pathway is closely related to apoptosis [32-34], the miR-4433a-5p inhibitor might inhibit the activity of the PI3K/Akt signaling pathway by upregulating the expression of the target gene *PIK3R2*, thereby inducing apoptosis in inflammatory cells.

Inevitably, this study also has some shortcomings that could be improved. First, this was a single-center study, and the number of clinical samples included in this study was limited. Relevant conclusions still need further confirmation by subsequent large-scale and multi-center prospective studies. In addition, based on the above results, we plan to build a clinical prediction model for the development of COPD. Finally, this study used A549 cells for *in vitro* experiments. We plan to build a mouse COPD model and conduct in-depth mechanism studies based on the model in our future studies.

## CONCLUSION

Preventive measures play a significant role in the clinical diagnosis and treatment of COPD. Biomarkers can predict the development and prognosis of the disease, provide a reference for the formulation of clinical treatment strategies, and improve the diagnosis and treatment effects of COPD. miR-4433a-5p, miR-1246, and miR-1290 might be markers and potential therapeutic targets for progressive COPD. At the same time, miR-4433a-5p might activate the PI3K/Akt signaling pathway, promote the proliferation of inflammatory A549 cells, inhibit cellular apoptosis, and further affect the progression of COPD disease by inhibiting the expression of PIK3R2.

## Figures and Tables

**Fig. (1):**
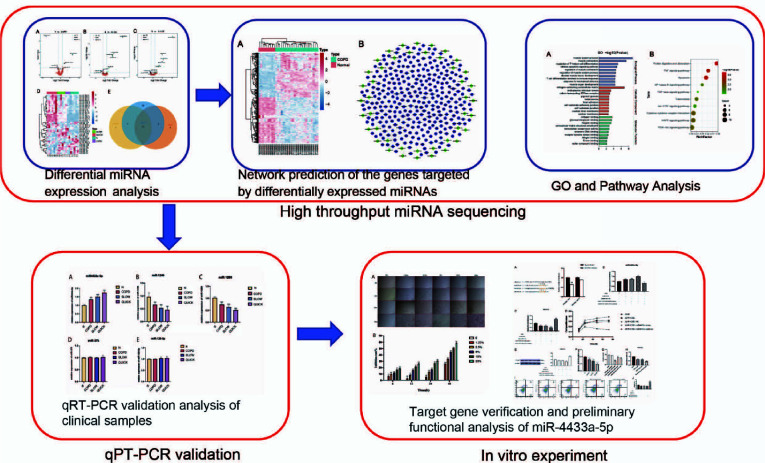
A flowchart of screening and mechanism of miRNA as a biomarker for patients with different progressive COPD. **Notes:** This research mainly includes three parts, which are as follows: High throughput miRNA sequencing、Target gene verification, and preliminary functional analysis of miR-4433a-5p、High throughput miRNA sequencing.

**Fig. (3):**
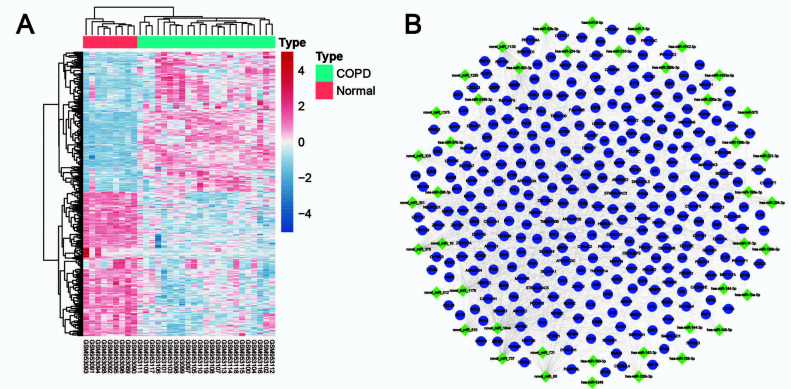
Network prediction and enrichment analysis of the genes targeted by differentially expressed miRNAs. **Notes: (A)** Heat map of differential expression between COPD and control groups; **(B)** Network prediction of the genes targeted by differentially expressed miRNAs. The green square represents miRNA; The purple circles represent miRNA target genes.

**Fig. (7):**
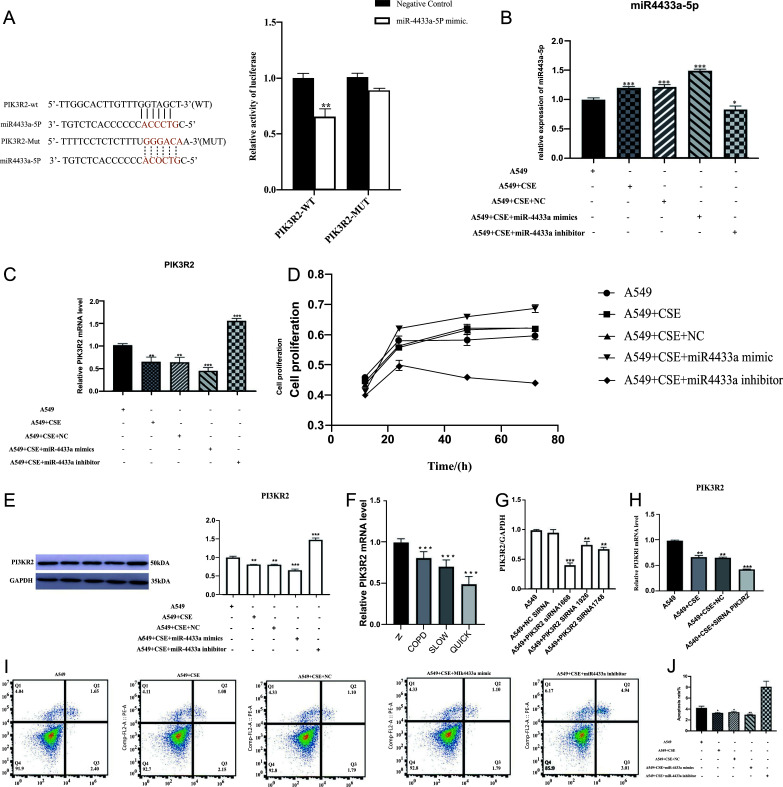
Target gene analysis and biological phenotype detection using dual-luciferase reporter assay and transfection test. **Notes:** (**A**) Dual-luciferase reporter assay results of miR-4433a-5p and PIK3R2. (**B**) Expression levels of miR-4433a-5p in the different transfection groups were detected using qRT-PCR. (**C**) Expression levels of PIK3R2 in the different transfection groups were detected using qRT-PCR. (**D**) Effects of miR-4433a-5p transfection on the proliferation of inflammatory cells. (**E**) Expression levels of PIK3R2 in the different transfection groups were detected using Western blot. (**F**) Expression levels of PIK3R2 in patients with “slow” and “fast” progression of COPD. (**G**) siRNA of optimized target sequence PIK3R2. (**H**) Expression levels in different transfection groups after knockdown of PIK3R2; (**I**) and **(J**) Apoptosis of A549 cells in the different transfection groups.

**Table 1:** Clinical data of patients enrolled.

**Group**	**Sample No.**	**Gender**	**Age**	**Smoking**
N_FAST	F1	male	48	Yes
	F2	male	62	Yes
	F3	male	66	Yes
	F4	male	66	Yes
	F5	male	46	Yes
	F6	male	68	Yes
N_SLOW	S1	male	63	Yes
	S2	male	73	Yes
	S3	male	64	Yes
	S4	male	56	Yes
	S5	male	60	Yes
COPD patients	Z1	male	56	Yes
	Z2	male	70	Yes
	Z3	male	63	Yes
Normal group	N1	male	54	Yes
	N2	male	53	Yes
	N3	male	72	Yes
	N4	male	54	Yes
	N5	male	47	Yes

## Data Availability

Not applicable.

## LIST OF ABBREVIATIONS

COPD: Chronic Obstructive Pulmonary Disease
OD: Optical Density
3’UTR: 3’ Untranslated Region
PF-ILD: Progressive Interstitial Lung Disease

## STANDARDS OF REPORTING

STROBE guidelines have been followed in this study.
